# Understanding the mechanisms of climate change impact on tuberculosis: a complex systems approach

**DOI:** 10.1186/s12889-025-24709-6

**Published:** 2025-10-08

**Authors:** Yahya Shadi, Esmaeil Khedmati Morasae, Salman Khazaei, Mahshid Nasehi, Saeed Sharafi, Hossein Asakereh, Leili Tapak, Zohreh Kahramfar, Younes Mohammadi

**Affiliations:** 1 Department of Epidemiology, School of Public Health, Hamadan University of Medical Sciences, Hamadan, Iran; 2 Department of Management Science, Business School, University of Strathclyde, Glasgow, Scotland UK; 3 Department of Epidemiology, Research Center for Health Sciences, Hamadan University of Medical Sciences, Hamadan, Iran; 4 Department of Epidemiology, Iran University of Medical Sciences, Tehran, Iran; 5 Center for Communicable Diseases Control, Ministry of Health and Medical Education, Tehran, Iran; 6 Department of Geography, University of Zanjan, Zanjan, Iran; 7 Department of Biostatistics, School of Public Health and Modeling of Noncommunicable Diseases Research Center, Hamadan University of Medical Sciences, Hamadan, Iran; 8 Department of Internal Medicine, School of Medicine, Hamadan University of Medical Sciences, Hamadan, Iran; 9 Social Determinants of Health Research Center, Hamadan University of Medical Sciences, Hamadan, Iran

**Keywords:** Climate change, Tuberculosis, Complex systems, Environmental determinants of health

## Abstract

**Background:**

Tuberculosis (TB) is a leading cause of disability and mortality in many countries and is the leading cause of death from an infectious agent worldwide. While TB is a curable and preventable disease, health systems’ ineffectiveness in case finding and appropriate treatment results in 10 million new cases and 1.5 million deaths annually around the globe. Climate change is expected to have a major impact on TB and other infectious diseases, although the mechanisms for this are still poorly understood.

**Methods:**

We undertook a systematic review of Literature published up to September 2024 about the effects of climate Change on TB incidence. The review identified 35 papers that described possible mechanisms for the impact of climate change on TB. We used a complex systems approach called causal loop diagramming to integrate the identified mechanisms into a system map of climate change effects on TB. A panel of experts on TB, epidemiology, and climate change reviewed the map’s structure and content.

**Results:**

The final map shows 6 reinforcing feedback loops and associated chains of complex bio-socio-technical interrelations through which climate change can affect TB risk. The loops included reciprocal relationships between heatwave − energy use, indoors time − airborne disease risk, food access − price, malnutrition − infectious disease, healthcare cost − detection delay, and infectious contact − TB risk that translate to TB infection, directly or indirectly, when activated.

**Conclusions:**

The presented map illustrates and highlights the need for coordinated, multisectoral and complex interventions across that bio-socio-technical system to tackle the nexus of climate change and TB risk. In this context, identifying key leverage points and implementing strategic actions on these points are essential to effectively mitigate climate change–related risks and their impact on TB transmission and incidence.

**Supplementary Information:**

The online version contains supplementary material available at 10.1186/s12889-025-24709-6.

## Introduction

Tuberculosis (TB) remains one of the top ten causes of death globally and is the leading cause of death from an infectious agent. Despite being a preventable and curable disease, TB continues to pose a significant public health challenge [1]. According to the World Health Organization (WHO), nearly 10 million people contract TB annually, and approximately 1.5 million people succumb to the disease each year. The United Nations Sustainable Development Goals (SDGs), particularly SDG 3, aim to end the global TB epidemic by 2030. In alignment, the Who End TB Strategy has set ambitious targets to reduce TB incidence by 90% and TB deaths by 95% by 2035 compared to 2015 levels [2]. However, global reports indicate that progress towards these targets has been slow and that this global challenge is not evenly distributed [3]. The burden of TB is disproportionately borne by Low- and middle-income countries, which account for over 80% of cases and deaths [4]. According to the 2023 Global TB Report, eight countries together accounted for approximately two-thirds of the global TB cases in 2022: India (27.0%), Indonesia (10.0%), China (7.1%), the Philippines (7.0%), Pakistan (5.7%), Nigeria (4.5%), the Democratic Republic of the Congo (4.5%), and Bangladesh (3.0%) [3].

Among healthy adults infected with TB bacteria, 5%–10% are at risk of developing active TB during their lifetimes, with the concomitant risks of TB morbidity, mortality, and transmission [4]. This risk is significantly higher among individuals of low socioeconomic status with compromised immune systems, such as those living with HIV, malnutrition, or diabetes [5]. This means that reducing the burden of TB in an effective way requires a broader approach than medical treatment alone. While biomedical interventions are essential, they are not sufficient on their own to bring an end to the TB epidemic. Indeed, decades of accumulated evidence show that social, economic, and structural factors play a critical role in the transmission and progression of TB [2, 6].Without an integrated response that addresses these underlying determinants, efforts to eliminate TB are unlikely to achieve their intended outcomes.

Climate change is now widely acknowledged as one of the most pressing health challenges of our time. It causes or exacerbates a range of issues, including extreme heat events, emergence and spread of new infectious diseases, wildfires, and depletion of natural resources, all identified as public health emergencies [7, 8]. Who estimates that by 2030, the direct health costs associated with climate Change could reach up to 2 to 4 billion dollars annually [9]. Alongside those social factors mentioned above, climate change also plays a crucial role in influencing the incidence and overall burden of TB [10, 11]. A growing body of research has explored the complex interactions between climatic factors and the occurrence of TB, highlighting the profound effects that environmental changes can have on public’s health. For instance, Khaliq et al. conducted a study in Lahore, Pakistan, spanning from 2006 to 2013, and found a significant correlation between temperature and the incidence of TB [12]. In a similar vein, Zhang et al. (2019) performed a systematic analysis of TB incidence in Beijing, utilizing structural equation modelling. Their research indicated of a positive relationship between increased precipitation, atmospheric pressure, relative humidity, and the incidence of TB [13]. Rao and colleagues (2016) employed a spatial panel data model to reveal a positive correlation between TB incidence and environmental factors such as high temperature, precipitation, and wind speed [14]. In Malaysia, Mohidem et al. (2021) identified significant associations between TB incidence and environmental factors, including carbon monoxide (CO), nitrogen dioxide (NO_2_), sulfur dioxide (SO_2_), inhalable particulates, rainfall, relative humidity, high temperature, wind speed, and atmospheric pressure [15]. Fernandes et al. (2017) also demonstrated a positive correlation between air humidity, high temperature, particulates, and TB incidence [16].

Any public health intervention aimed at improving a health condition (e.g. TB incidence) is grounded in a particular understanding of the underlying causal structure of that condition. The more accurate and precise this understanding, the greater the likelihood of developing effective solutions and interventions to enhance population health. Systems science, an interdisciplinary field with a holistic perspective, seeks to provide a deeper understanding of the nature and causal structures of the bio-socio-technical systems that surround us [17, 18]. Systems scientists employ systemic tools to explore the dynamics of complex, multifaceted phenomena over time, while accounting for the role of interacting feedback loops and causal relationships within these systems [19]. This approach facilitates the design of coordinated, system-wide interventions with the goal of shifting a system's trajectory toward more favourable outcomes. This systemic perspective is increasingly gaining recognition in public health literature, emerging as an epistemic standard for research and practice [20, 21].

As mentioned, TB is a complex phenomenon influenced by a wide range of bio-socio-technical factors, including malnutrition, smoking, HIV/AIDS, diabetes, poverty, and access to diagnostic, treatment, and care services [22]. Similarly, climate change is a complex and multifaceted global issue [23, 24]. These two complex challenges are now increasingly intersecting and compounding each other’s effects. We believe that understanding the full scope of this complexity, and addressing the human costs associated with it, requires an approach rooted in systems thinking. To be precise, such complex public health challenges cannot be addressed through traditional, reductionist methods that focus primarily on linear cause-and-effect relationships. These approaches often isolate variables and assume stability over time, making them ill-equipped to capture the dynamic interactions, feedback loops, and adaptive behaviors inherent in systems like climate change and TB transmission. As a result, they overlook crucial mechanisms such as cumulative effects, unintended consequences, and reinforcing vulnerabilities—gaps that complex systems approaches are specifically designed to address [25]. This complexity is one of the reasons behind calls for deeper investigation of climate change impact on TB that is far from being well-understood at this moment [12–16].

In this study, as a result, we adopted a complex systems approach to investigate the mechanisms through which climate change may influence TB. We began by conducting a systematic review of the literature to identify the relevant mechanisms. Using these insights, we then employed a causal loop diagramming approach to develop a system map that synthesizes and represents these mechanisms holistically, offering a comprehensive view of how climate change impacts TB.

## Methods

### Eligibility criteria

Duplicate articles were identified and removed using EndNote software. Subsequently, two independent reviewers (YS and EKM) screened the titles and abstracts of the remaining studies, excluding those that did not meet the inclusion criteria. We included all English-language studies that explored and reported the impact mechanisms of climate change on TB, irrespective of geographical region. Full texts of potentially relevant studies were then retrieved for further detailed evaluation by YS and EKM.

### Information sources

We performed a comprehensive search across three major databases—Web of Science, PubMed, and Scopus—covering studies published until September 2024. Additionally, we manually reviewed the reference lists of the included studies to identify any further relevant articles.

### Search strategy

The search strategy combined the following terms: "climate change," "global climate changes," "weather changes," and "global warming" with "communicable diseases," "infectious diseases," "contagious diseases," "transmissible diseases," "Mycobacterium tuberculosis," "tuberculosis," "M. tuberculosis," and "TB." (Please see Additional file 1, Supplementary Table 1 for detailed search strategy).

### Data extraction

A structured codebook was developed to systematically capture the mechanisms described across the included studies. Key details—such as publication year, author(s), variables involved, the nature of their relationships, and the directionality of those relationships—were extracted and registered in the codebook. Any discrepancies between the two primary reviewers were discussed and resolved in consultation with a third researcher (YM) during a consensus meeting.

Since our study aimed to investigate the mechanisms through which climate change affects tuberculosis, and not to quantify the effect sizes, there was no need to prepare additional data given the qualitative nature of the synthesis through system mapping and categorization. Accordingly, sensitivity analysis was not applicable.

### System mapping

We adopted a complex systems approach to explore the complex pathways through which climate change influences TB. Complex systems thinking focuses on how interconnected elements interact over time, often in non-linear and feedback-driven ways. Rather than isolating single causes, this approach reveals how multiple factors dynamically influence TB outcomes [26].

To conceptualize these relationships and interactions, we used system mapping, a method that visually represents how different variables are linked and influence one another. Specifically, we developed a Causal Loop Diagram (CLD), a widely used tool for identifying reinforcing and balancing feedback loops in complex systems. Each CLD consists of nodes (key variables), connections (causal relationships), and feedback loops (circular interactions that either amplify or stabilize change) [27]. The structured codebook guided the development of our CLD. Using Vensim software, we progressively translated the reported variables and their relationships into a visual system map, reflecting the dynamic nature of the problem. This CLD offers a novel, structured view of how environmental, social, and biological factors interact to shape TB outcomes under climate change pressures. Unlike traditional linear models, it captures the dynamic feedbacks and cascading effects that can exacerbate or mitigate TB transmission over time. This systems-based perspective is particularly valuable for identifying leverage points where policy interventions—such as housing, air quality, or healthcare access—could produce broader TB benefits. By making complex interdependencies visible, this CLD serves not only as a conceptual map but also as a practical decision-support tool for researchers, policymakers, and public health professionals seeking to design more integrated and forward-looking responses to climate-induced TB risks [27, 28].

### Validation of the CLD

The constructed CLD was then presented to a panel of experts specializing in TB and climate Change, consisting of 12 participants, during a one-day validation workshop. The workshop, facilitated by YS, EKM, and YM, brought together a diverse group of professionals, including epidemiologists, respiratory specialists, climatologists, TB care providers, and national TB control managers from Iran. The professionals were selected based on predefined criteria, including practical experience in the care and treatment of TB, a minimum of five years of experience in TB epidemiology and the national TB surveillance system in Iran, academic expertise in the impact of climate change on health, prior participation in national or international TB related research projects, and relevant publications in reputable journals. The primary objective was to validate the findings and ensure the CLD accurately reflected the current scientific understanding of the relationship between climate change and TB. During the workshop, each link in the CLD was meticulously reviewed and subjected to in-depth discussion to verify both its content and directional flow. The panelists were largely in agreement with the overall structure of the CLD. However, a few modifications were suggested, particularly in terms of the naming and description of specific relationships, to improve clarity and ensure the map was easily interpretable by a broad audience. In addition, some intermediary variables were introduced to further refine the connections between key elements, providing a more detailed representation of the pathways involved. These adjustments enhanced the overall coherence and depth of the CLD, ensuring it effectively captured the complex interactions between climate change and TB transmission.

## Results

**Fig. 1:**
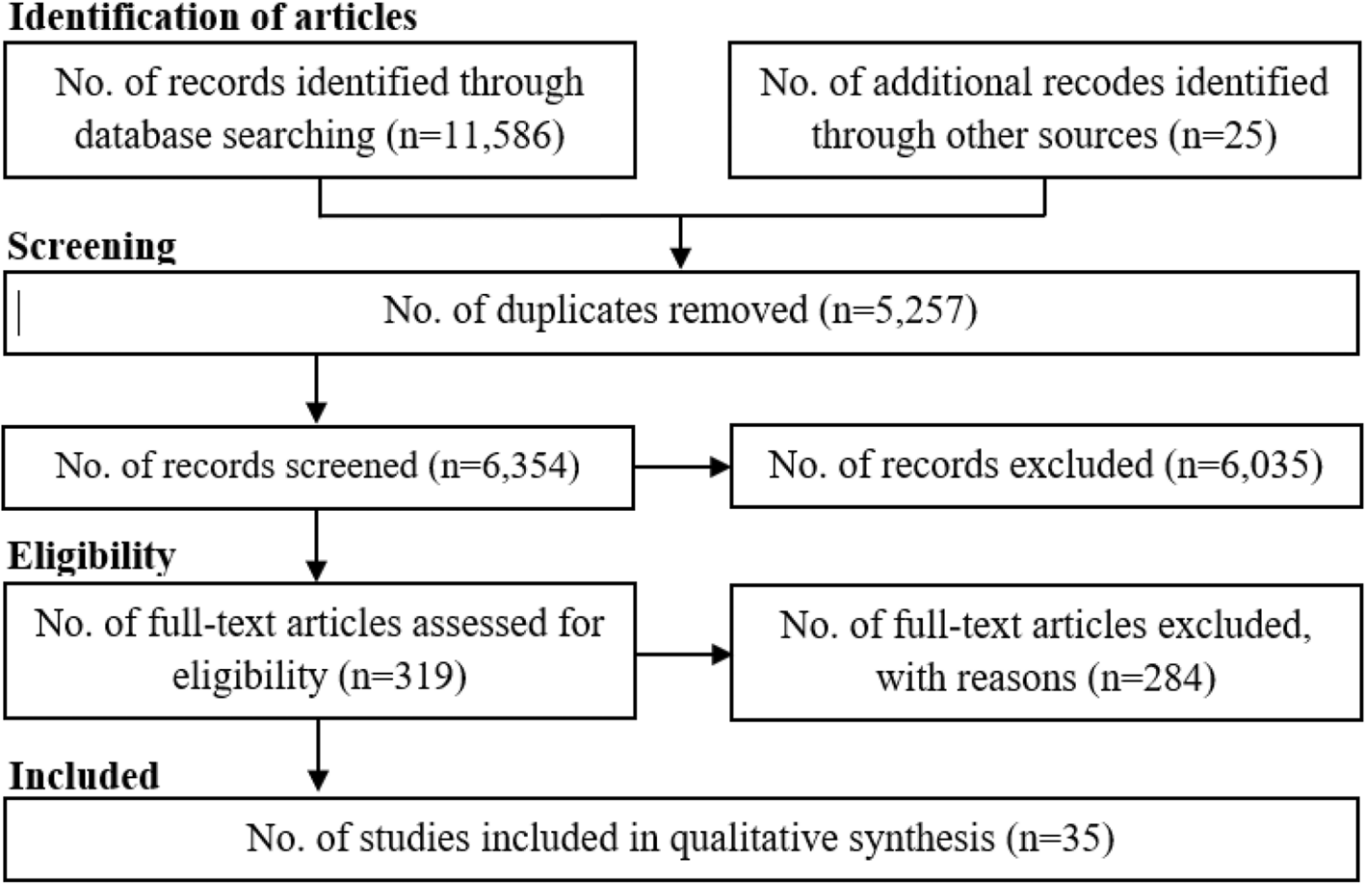
Steps taken in the systematic review process

A total of 11,586 articles were initially retrieved through our research strategy. The titles of the retrieved articles were screened by YS and EKM to identify studies most relevant for further investigation. As a result, 319 articles were selected for abstract screening to assess whether they met the inclusion criteria. Any discrepancies between the researchers during these two stages were discussed and resolved in two subsequent meetings with YM. Following this process, 35 articles were ultimately chosen for full-text analysis and data extraction [12–14, 16, 29–59]. YS conducted the data extraction, while EKM and YM oversaw the process to ensure that all necessary information was accurately collected and recorded from the final pool of papers (Fig. 1).

Articles published after 2015 demonstrate an increasing trend in attention to climate change implications for TB, highlighting the growing importance of this topic and the rising interest of researchers in the issue. All identified mechanisms are summarized in Table 1.

**Table 1:** A summary of articles reviewed, and mechanisms identified*

Author	Year	Country/region	Mechanism
Rio’s M. et al. [51]	2000	Spain	Climate change → Heat and cold wave likelihood ↑ (a) Average time spent indoors due to extreme heat or cold ↑ Vitamin D synthesis ↓ Strength of immune system ↓ Tuberculosis risk ↑
			(b)Temporary housing ↑ Population density ↑ Ventilation rate↓ Likelihood of airborne diseases risk ↑Mycobacterium shedding rate ↑ Infectious TB contact ↑ Tuberculosis risk ↑
Kremer L. et al. [43]	2002	UK	Climate change → Heatwave likelihood ↑ (a) Fluidity of Macrobacterium membrane lipids ↑ Mycobacterium permeability rate ↑ Mycobacterium persistence rate ↑ Infectious TB contact ↑ Tuberculosis risk ↑
Shilova M.V. et al. [53]	2004	Russia	Climate change → (a) Atmospheric SO2 levels ↑ Alveolar macrophages count ↓ Immune system strength ↓ Tuberculosis risk ↑
			(b) Atmospheric NO2 levels ↑ Blood monocytes count ↓ Immune system strength ↓ Tuberculosis risk ↑
Habib R.R. et al. [39]	2010	EMRO	Climate change → (a) Likelihood of extreme temperature events ↑ Likelihood of temporary housing ↑ Population density ↑ Environmental health conditions ↓ malnutrition risk ↑ Tuberculosis risk ↑
Grassly NC et al. [37]	2011	Global	Climate change → Heatwave likelihood ↑ (a) Average time spent indoors ↑ Population density ↑ Ventilation rate↓ Infectious TB contact ↑ Tuberculosis risk ↑
			(b) Likelihood of torrential rains ↑ Atmospheric humidity level ↑ Mycobacterium growth rate ↑ Infectious TB contact ↑ Tuberculosis risk ↑
			(c) Average number of sunny days ↑ UV radiation level ↑ Mycobacterium growth rate ↓ Infectious TB contact ↓ Tuberculosis risk ↓
			(d) Humidity level ↓ Respiratory tracts mucus level ↓ Respiratory tract susceptibility to infection↑ Tuberculosis risk ↑
Fares A. et al. [36]	2012	Global	Climate change → a) Heat and cold wave likelihood ↑ Average time spent indoors ↑ Vitamin D synthesis ↓ Strength of immune system ↓ Tuberculosis risk ↑
			(b) Repeated heatwave likelihood ↑ Geographical range of respiratory pathogens ↑ New respiratory diseases likelihood↑ Mycobacterium shedding rate ↑ Infectious TB contact ↑ Tuberculosis risk ↑
			(c) Heatwave likelihood ↑ Fluidity of Macrobacterium membrane lipids ↑ Mycobacterium permeability rate ↑ Mycobacterium persistence rate ↑ Infectious TB contact ↑ Tuberculosis risk ↑
Butler CD. et al. [30]	2012	Global	Climate change → Heatwave likelihood ↑ a) Drought likelihood ↑ Food Production ↓ Food access ↓ Malnutrition risk ↑ Strength of immune system ↓ Tuberculosis risk ↑
			b) Drought likelihood ↑ Food Production ↓ Food prices ↑ Malnutrition risk ↑ Strength of immune system ↓ Tuberculosis risk ↑
			c) Tuberculosis risk↑ Work capacity (of patients) ↓ Healthcare cost affordability ↓ Time to detection ↑ infectious contact risk ↑
			d) Food prices ↑ Healthcare cost affordability ↓ Time to detection ↑ infectious contact risk ↑
			e) Drought likelihood ↑ Food Production ↓ Food prices ↑ Malnutrition risk ↑ Strength of immune system ↓ Susceptibility to infectious diseases ↑ Nutrients absorption level ↓ Tuberculosis risk ↑
			f) Food production ↑ Food prices ↓ Food access ↑
			g) Food production ↑ Food Access ↑ Food prices ↓
Hansen J, et al. [40]	2012	USA	Climate change → a) Heatwave likelihood ↑ Drought likelihood ↑ Food Production ↓ Food access ↓ Malnutrition risk ↑ Strength of immune system ↓ Tuberculosis risk ↑
			b) Heatwave likelihood ↑ Drought likelihood ↑ Food Production ↓ Food prices ↑ Malnutrition risk ↑ Strength of immune system ↓ Tuberculosis risk ↑
			c) Likelihood of torrential rains ↑ Atmospheric humidity level ↑ Mycobacterium growth rate ↑ Infectious TB contact ↑ Tuberculosis risk ↑
Davis LW. et al. [33]	2014	MEXICO	Climate change → (a) Heatwave likelihood ↑ Air conditioner use rate ↑ Electricity consumption rate ↑CO2 atmospheric level ↑ Heatwave likelihood
Onozuka D. et al. [49]	2014	Japan	Climate change → Heatwave and cold wave likelihood ↑ (a) Average time spent indoors ↑ Vitamin D synthesis ↓ Strength of immune system ↓ Tuberculosis risk ↑
			Climate change → Dust pollution level ↑ (a) Average time spent indoors ↑ Population density ↑ Ventilation rate ↓ Infectious TB contact ↑ Tuberculosis risk ↑
Khaliq A, et al. [12]	2015	Pakistan	Climate change → Heatwave and cold wave likelihood ↑ (a) Average time spent indoors ↑ Vitamin D synthesis ↓ Strength of immune system ↓ Tuberculosis risk ↑
Lai TC, et al. [44]	2015	Taiwan	Climate change → (a) Atmospheric NO2 levels ↑ Alveolar macrophages count/Blood monocytes count ↓ Immune system strength ↓ Tuberculosis risk ↑
Smith GS. et al. [54]	2015	USA	Climate change → (a) Particulate matter emission level↑ Respiratory tract susceptibility to infection ↑ Tuberculosis risk ↑
Álvaro-Meca A. et al. [29]	2016	Spanish	Climate change → (a) Heatwave and cold wave likelihood ↑ Average time spent indoors ↑ Vitamin D synthesis ↓ Strength of immune system ↓ Tuberculosis risk ↑ b) NO2/SO2/CO/O3/Particulate matter atmospheric level ↑ Oxidative stress level↑ Immune system strength ↓ Tuberculosis risk ↑
Perova OB. et al. [50]	2016	Russia	Climate change → Heatwave and cold wave likelihood ↑ (a) Average time spent indoors ↑ Vitamin D synthesis ↓ Strength of immune system ↓ Tuberculosis risk ↑
			b) Average time spent indoors ↑ Population density ↑ Ventilation rate ↓ Infectious TB contact ↑ Tuberculosis risk ↑
Fernandes FM. et al. [16]	2016	Brazil	Climate change → Heatwave and cold wave likelihood ↑ (a) Average time spent indoors ↑ Vitamin D synthesis ↓ Strength of immune system ↓ Tuberculosis risk ↑
			(b) Likelihood of torrential rains ↑ Atmospheric humidity level ↑ Mycobacterium growth rate ↑ Infectious TB contact ↑ Tuberculosis risk ↑
Smith GS. et al. [55]	2016	USA	Climate change → (a) NO2/SO2 atmospheric level ↑ Alveolar macrophages count/Blood monocytes count ↓ Respiratory tract inflammation level↑ Strength of immune system ↓ Tuberculosis risk ↑
			(b) CO/O3 atmospheric level ↑ Respiratory tract inflammation level↑ Strength of immune system ↓ Tuberculosis risk ↑
Rao HX. et al. [14]	2016	China	Climate change → Heatwave and cold wave likelihood ↑ (a) Average time spent indoors ↑ Vitamin D synthesis ↓ Strength of immune system ↓ Tuberculosis risk ↑
			b) Average time spent indoors ↑ Population density ↑ Ventilation rate ↓ Infectious TB contact ↑ Tuberculosis risk ↑
Nardell EA. [48]	2016	-	Climate change → a) Heatwave likelihood ↑ Air conditioner use rate ↑ Ventilation rate ↑ infectious TB contact ↓ Tuberculosis risk ↓
Guo C. et al. [38]	2017	China	Climate change → Heatwave and cold wave likelihood ↑ (a) Average time spent indoor ↑ Vitamin D synthesis ↓ Strength of immune system ↓ Tuberculosis risk ↑
			(b) Likelihood of torrential rains ↑ Atmospheric humidity level ↑ Mycobacterium growth rate ↑ Infectious TB contact ↑ Tuberculosis risk ↑
Zhang Y. et al. [58]	2019	China	Climate change → a) Heatwave likelihood ↑ Likelihood of torrential rains ↑ Atmospheric humidity level ↑ Mycobacterium growth and persistence rate ↑ Infectious TB contact ↑ Tuberculosis risk ↑
			b) Average number of sunny days ↑ UV radiation level ↑ Mycobacterium growth rate ↓ Infectious TB contact ↓ Tuberculosis risk ↓
			c) Wind speed ↑Mycobacterium environmental concentration ↓ Infection transmission risk ↓ Tuberculosis risk ↓
Sarkar S. et al. [52]	2019	Mexico	Climate change → Heatwave and cold wave likelihood ↑ (a) Average time spent indoors ↑ Vitamin D synthesis ↓ Strength of immune system ↓ Tuberculosis risk ↑
			(b)Temporary housing ↑ Population density ↑ Ventilation rate↓ Airborne diseases risk ↑Mycobacterium shedding rate ↑ Infectious TB contact ↑ Tuberculosis risk↑
			c) Heatwave likelihood ↑ NO2/SO2 atmospheric level ↑ Alveolar macrophages count/Blood monocytes count ↓ Respiratory tract inflammation level↑ Strength of immune system ↓ Tuberculosis risk ↑
Zhang CY. et al. [13]	2019	China	Climate change → a) Particulate matter emission level ↑ Microorganism lung penetration level ↑ Tuberculosis risk ↑
			b) Wind speed ↑ Mycobacterium translocation rate ↑Infection transmission risk ↑ Tuberculosis risk ↑
			c) Heatwave likelihood ↑ NO2/SO2 atmospheric level ↑ Alveolar macrophages count/Blood monocytes count ↓ Respiratory tract inflammation level↑ Strength of immune system ↓ Tuberculosis risk ↑
			d) Dust pollution level ↑ Respiratory immune response intensity ↑ Respiratory tract susceptibility to infection ↑ Tuberculosis risk ↑
Jaganath D. et al. [41]	2019	Uganda	Climate change → Heatwave likelihood ↑ (a) Average time spent indoors ↑ Population density ↑ Ventilation rate ↓ Infectious TB contact ↑ Tuberculosis risk ↑
			(b) Likelihood of torrential rains ↑ Atmospheric humidity level ↑ Mycobacterium growth rate ↑ Infectious TB contact ↑ Tuberculosis risk ↑
			c) Average time spent indoor ↑ Vitamin D synthesis ↓ Strength of immune system ↓ Tuberculosis risk ↑
			d) Temporary housing ↑ Population density ↑ Ventilation rate↓ Airborne diseases risk ↑Mycobacterium shedding rate ↑ Infectious TB contact ↑ Tuberculosis risk↑
Yao L. et al. [57]	2019	China	Climate change → a) Particulate matter emission level ↑ Mycobacterium lung penetration level ↑ Tuberculosis risk ↑
			b) Heatwave likelihood ↑ NO2/SO2 atmospheric level ↑ Alveolar macrophages count/Blood monocytes count ↓ Respiratory tract inflammation level↑ Strength of immune system ↓ Tuberculosis risk ↑
Yang J. et al. [56]	2020	China	Climate change → a) Particulate matter emission level ↑ Mycobacterium lung penetration level ↑ Tuberculosis risk ↑
			b) Heat and cold wave likelihood ↑ Average time spent indoors ↑ Population density ↑ Ventilation rate ↓ Infectious TB contact ↑ Tuberculosis risk ↑
			(c) Atmospheric SO2 levels ↑ Alveolar macrophages count ↓ Oxidative stress level ↑ Immune system strength ↓ Tuberculosis risk ↑
			(d) Atmospheric NO2 levels ↑ Blood monocytes count ↓ Oxidative stress level ↑ Immune system strength ↓ Tuberculosis risk ↑
			e) CO atmospheric level ↑ Respiratory tract inflammation level ↑ Oxidative stress rate ↑ Immune system strength ↓ Tuberculosis risk ↑
Li ZQ. et al. [46]	2020	China	Climate change → a) Heat cold wave likelihood ↑ Average time spent indoor ↑ Population density ↑ Ventilation rate ↓ Infectious TB contact ↑ Tuberculosis risk ↑
			b) Wind speed ↑ Natural ventilation ↑ Infection transmission risk ↓ Tuberculosis risk ↓
			c) Likelihood of torrential rains ↑ Atmospheric humidity level ↑ Mycobacterium growth and persistence rate ↑ Infections TB contact ↑ Tuberculosis risk ↑
Zheng Y. et al. [59]	2020	China	Climate change → a) Heat and cold wave likelihood ↑ Average time spent indoor ↑ Population density ↑ Ventilation rate ↓ Infectious TB contact ↑ Tuberculosis risk ↑
			b) O3 atmospheric level ↑ Respiratory tract inflammation level ↑ Immune system strength ↓ Tuberculosis risk ↑
Fanzo JC. et al. [35]	2021	-	Climate change → a) Heatwave likelihood ↑ Drought likelihood ↑ Food Production ↓ Food access ↓ Malnutrition risk ↑ Strength of immune system ↓ Tuberculosis risk ↑
			b) Heatwave likelihood ↑ Drought likelihood ↑ Food Production ↓ Food prices ↑ Malnutrition risk ↑ Strength of immune system ↓ Tuberculosis risk ↑
Chen D. et al. [32]	2021	China	Climate change → a) Heatwave likelihood ↑ Average number of sunny days ↑ UV radiation level ↑ Mycobacterium growth rate ↓ Infectious TB contact ↓ Tuberculosis risk ↓
			b) Heatwave likelihood ↑ Air conditioner use rate ↑ Ventilation rate ↑ infectious TB contact ↓ Tuberculosis risk ↓
			c) Average time spent indoor ↑ Vitamin D synthesis ↓ Strength of immune system ↓ Tuberculosis risk ↑
Kirolos A. et al. [42]	2021	Malawi	Climate change → a) Heatwave likelihood ↑ Drought likelihood ↑ Poverty level ↑ Emigration level ↑ Urban marginalization rate↑ Housing quality ↓ Ventilation rate ↓ Infectious contact risk↑ Infection transmission risk ↑ Tuberculosis risk ↑
			b) Heatwave likelihood ↑ Drought likelihood ↑ Poverty level ↑ Emigration level ↑ Urban marginalization rate↑ HIV infection risk ↑ Strength of immune system ↓ Tuberculosis risk ↑
			c) Dust pollution level↑ Drought likelihood ↑ Poverty level ↑ Emigration level ↑ Urban marginalization rate↑ HIV infection risk ↑ Strength of immune system ↓ Tuberculosis risk ↑
Li H. et al. [45]	2022	China	Climate change → O3 atmospheric level ↑Respiratory tract inflammation level ↑ Oxidative stress level ↑ Immune system strength ↓ Tuberculosis risk ↑
Di Napoli. et al. [34]	2023	Global	Climate change → a) Heatwave likelihood ↑ Likelihood of torrential rains ↑ Soil erosion rate ↑ Land fertility level ↓ Food Production ↓ Food access ↓ Malnutrition risk ↑ Strength of immune system ↓ Tuberculosis risk ↑ Tuberculosis risk ↑
Chang M. et al. [31]	2024	China	Climate change → Wind speed ↑ Mycobacterium translocation rate ↑Infection transmission risk ↑ Tuberculosis risk ↑
Makrufardi F. et al. [47]	2024	Taipei	Climate change → a) Particulate matter emission level ↑ Mycobacterium lung penetration level ↑ Tuberculosis risk ↑
			b) Heatwave likelihood ↑ NO2/SO2 atmospheric level ↑ Alveolar macrophages count/Blood monocytes count ↓ Respiratory tract inflammation level↑ Strength of immune system ↓ Tuberculosis risk ↑

^*^(→ means leads to; ↑ means increase; ↓ means decrease)

In consultation with experts who validated the constructed CLD, we categorized the identified mechanisms into the following five groups. The categories were thought to be illustrative enough to summarize the system map content succinctly:

1. Malnutrition
2. Air quality and pollution
3. Extreme temperature events (e.g. heatwaves and cold spells)
4. Airborne diseases epidemics
5. Peripheral mechanisms

According to the reviewed literature, comparatively speaking, malnutrition appears to have the greatest impact on TB risk (HR = 2.23) [60], while air pollution (OR = 1.50) [55], extreme temperature fluctuations (RR ~ 1.2) [49], and rainfall (RR = 1.32) [61]. also contribute substantially.

### Malnutrition

Studies suggest that global warming will lead to rising sea surface temperatures, increasing the likelihood of torrential rains (Table 1). These heavy rains and subsequent floods are expected to intensify soil erosion, putting the agricultural and food production industries at risk. This can result in higher rates of malnutrition, a well-known factor associated with increased susceptibility to TB [34]. In fact, research shows that irregular rainfall and extreme temperature events negatively impact both the quantity and quality of agricultural products, leading to greater food insecurity and financial instability—two key precursors to malnutrition [30, 34, 40]. Additionally, individuals displaced by floods are more likely to live in temporary, overcrowded shelters, and the risk of respiratory diseases, including TB, is heightened [39]. The combination of malnutrition, food insecurity, and poor living conditions significantly increases vulnerability to TB.

### Air quality and pollution

Climate change can negatively impact air quality by increasing the concentration of inhalable particulate matter (those with a diameter of 2.5 to 10 microns) in the atmosphere (Table 1). Elevated PM levels facilitate the adhesion of microorganisms to these particles, enhancing the penetration of Mycobacterium deep into lung tissue [29, 47, 54, 56–58]. Additionally, exposure to PM has been shown to damage the respiratory system’s immune response [13, 29]. Similarly, higher levels of SO₂ in the environment can increase the risk of developing TB, as SO₂ can destroy alveolar macrophages and reduce immunologically active substances in the body [13, 29, 47, 52, 53, 55–57]. NO₂ can lead to similar outcomes, with studies showing that elevated NO₂ levels weaken the immune system by inhibiting the formation of neutralizing antibodies and disrupting the phagocytosis process in alveolar macrophages and blood mononuclear cells [13, 29, 44, 47, 52, 53, 55–57]. CO exposure has been shown to inhibit the immune response and exert inflammatory effects on the body [29, 55, 56]. Additionally, an increase in ozone (O₃) concentration in the atmosphere can severely irritate the respiratory tract, causing symptoms such as sore throat, chest tightness, coughing, bronchitis, and emphysema (that make the respiratory system more susceptible to TB infection), while also weakening the immune system [29, 45, 55, 59]. These environmental pollutants further weaken the immune system by inducing oxidative stress [29, 45, 56].

Oxidative stress occurs when there is an imbalance between the production of reactive oxygen species (ROS) (free radicals) and the body's ability to neutralize or detoxify their harmful effects using antioxidants. When ROS levels exceed the body’s antioxidant defenses, oxidative stress damages cells, proteins, DNA, and lipids, all of which compromise the immune system and increase the risk of developing TB [62].

### Heatwaves and cold spells

Heatwaves and cold spells can impact TB transmission through various mechanisms (Table 1). Extreme temperatures increase the amount of time people spend indoors, raising the likelihood of healthy individuals coming into contact with TB-positive individuals due to higher population densities in enclosed spaces. Factors such as inadequate ventilation and impaired vitamin D production—essential for immune system function—can further exacerbate TB transmission in these settings [12, 14, 16, 29, 32, 37, 38, 41, 46, 49, 50, 52, 59]. Additionally, extreme heat can raise surface water temperatures and increase evaporation, leading to a higher likelihood of torrential rains. Floods and heavy rainfall can indirectly influence TB by increasing environmental humidity (which allows droplet nuclei containing Mycobacterium tuberculosis to remain suspended in the air for longer periods) [31, 63], crowding in temporary shelters, and damaging healthcare infrastructure [16, 37, 38, 40, 41, 46, 58]. Moreover, extreme temperatures can alter the cell wall of Mycobacterium tuberculosis, allowing it to survive in harsh environmental conditions such as dehydration or elevated ambient temperatures, prolonging its survival in the environment [36, 43]. Collectively, these factors increase TB transmission and incidence rates.

### Epidemics of air borne diseases

A warmer climate can create favorable environmental conditions for the (re)introduction, growth, and reproduction of microorganisms across various geographical areas, leading to the spread of infectious respiratory diseases into new regions [64, 65]. The reviewed literature suggests that repeated climatic events, coupled with increased population density in temporary shelters (where people may remain for extended periods), inadequate ventilation, and limited healthcare facilities, provide ideal conditions for the easier transmission of airborne diseases and their subsequent outbreaks. These diseases, in turn, contribute to increased shedding and environmental load of Mycobacterium tuberculosis through frequent and severe coughing, making TB transmission and incidence more likely [39, 41, 51, 52] (Table 1).

### Peripheral mechanisms

Some scholars argue that mechanisms that initially seem unrelated or peripheral to TB may actually play a crucial role in shaping the future trends of the disease. For instance, while it is accepted that improved ventilation in indoor spaces can reduce the risk of TB transmission (although shown otherwise in some studies) [48], the use of mechanical ventilation systems also increases energy consumption and CO₂ emissions, exacerbating climate change. This, in turn, leads to more frequent heatwaves and an even greater demand for ventilation. Therefore, in the relationship between climate change and TB, ventilation can simultaneously act as both a solution and a contributor to the problem, complicating efforts to address the issue [33].

Another peripheral mechanism highlighted in the studies is climate-driven migration. Such migration, typically affecting impoverished populations or those rendered poor by climate events (e.g., droughts), often results in migrants settling in urban slums or marginalized areas. These environments are highly conducive to TB transmission, due to factors like overcrowded, low-quality housing and the higher prevalence of HIV, a condition that significantly compromises the immune system and often coexists with TB [42].

Interestingly, some scholars have also explored potential mitigating effects of climate change on TB trends. For example, it has been suggested that climate change could lead to increased wind speeds in certain regions, potentially improving natural ventilation and reducing the concentration of Mycobacterium in the environment [58]. Additionally, while heatwaves can cause torrential rains and increase indoor crowding, they also tend to increase the number of sunny days and elevate ultraviolet (UV) light exposure in some areas. A long sunshine duration with large amounts of UV light can restrict the development of M. tuberculosis. Moreover, UV exposure is the primary source of vitamin D production, which is essential for maintaining a strong immune system [32, 37, 58].

### A system map of the mechanisms

**Fig. 2:**
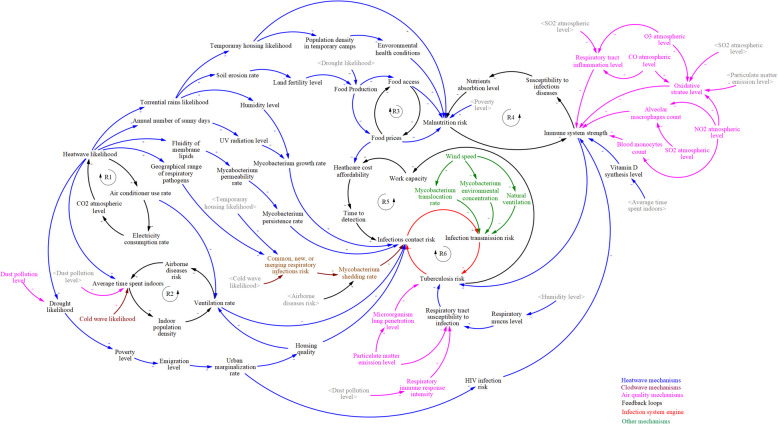
A system map of climate change effects on TB

We translated the factors and relationships outlined in Table 1 into a system map that displays the interactions among climate mechanisms and their related feedback loops (Fig. 2). Two mechanisms are briefly described as follows:

- Climate change increases the frequency and likelihood of heatwaves, which in turn leads to more torrential rainfall, often causing soil erosion. Soil erosion reduces land fertility and negatively impacts food production. Lower food production levels can affect food accessibility and drive-up prices, increasing the risk of malnutrition. Malnutrition weakens the immune system, making individuals more susceptible to TB and other infectious diseases, which can further disrupt nutrient absorption, worsening the malnutrition cycle. TB infection and activation reduce work capacity among affected individuals, who increasingly face unaffordable healthcare costs due to their inability to work. This often delays early detection and treatment of TB, increasing the chances of infectious contact and transmission to others.
- Climate change-induced heatwaves also increase the likelihood of droughts, leading to higher poverty levels. This economic hardship pushes people to migrate to the outskirts of cities, where housing quality is poor, and the risk of HIV is higher. Both these factors—compromised immune systems and TB-friendly environments—heighten the risk of TB infection.

In the system map, identified mechanisms and feedback loops are color-coded as follows: heatwave-related pathways in blue, cold-wave-related pathways in brown, air quality pathways in pink, other mechanisms in green, the first five feedback loops in black, and the system engine in red.

### Feedback loops

Feedback loops are key features of complex systems that help us understand their behavior more comprehensively. In the system map developed in this study, we identified six reinforcing feedback loops, each of which plays a crucial role in the TB-climate relationship (Table 2):

- R1: As heatwaves become more frequent, people increasingly rely on cooling devices to cope with rising temperatures. This greater dependence on cooling devices leads to higher energy consumption globally. Since much of this energy is generated by burning fossil fuels, CO₂ emissions rise, further contributing to global warming. This establishes a reinforcing feedback loop within the eco-social system, where rising temperatures drive higher energy use, more CO₂ emissions, and accelerated climate change. This feedback loop influences various interconnected pathways within the TB-climate system, ultimately heightening the risk of TB (as captured in subsequent loops).
- R2: With the increased likelihood of heatwaves, cold spells, and dust storms, the number of days people spend indoors rises, leading to greater population density in enclosed spaces. As indoor spaces become more crowded, ventilation rates decrease, heightening the risk of respiratory diseases. These diseases often necessitate home rest, which may be reinforced by quarantine and isolation measures. As a result, the time spent indoors continues to rise, creating another reinforcing feedback loop. Both reduced ventilation and higher rates of respiratory disease are well-known factors that contribute to the spread of TB.
- R3: The increased occurrence of heatwaves also raises the probability of torrential rainfall and flash floods. Flooding leads to soil erosion and decreased land fertility, resulting in lower agricultural output and reduced food availability. Food shortages drive up prices, making adequate nutrition harder to access. As the imbalance between supply and demand intensifies, rising food costs further limit stable access to food, creating a reinforcing feedback loop. This loop exacerbates the risk of malnutrition, which weakens the immune system and increases vulnerability to TB infection.
- R4: Several pathways in the climate-TB system increase the risk of malnutrition, which compromises the immune system. A weakened immune system heightens susceptibility to infectious diseases, which can, in turn, cause nutrient malabsorption, further worsening malnutrition. This creates a reinforcing feedback loop where infectious diseases both result from and contribute to malnutrition, thereby increasing the risk of TB infection and transmission.
- R5: TB is a leading cause of disability [66, 67], reducing work capacity and income. This loss of income diminishes people's ability to afford healthcare, delaying the early detection and treatment of TB. Delayed treatment increases the likelihood of infectious contact and the transmission of TB, further worsening the situation for both the affected individual and others in similar financial or social circumstances. The literature also suggests that rising food prices due to climate change could intensify this feedback loop, as healthcare becomes even more unaffordable for those struggling to meet basic needs.
- R6: This final reinforcing feedback loop serves as the engine or core of the system map. It illustrates that any mechanism that increases (or decreases) infectious contact with Mycobacterium results in higher (or lower) TB transmission rates, and consequently, an elevated (or reduced) risk of developing active TB. As active TB cases rise (or fall), the prevalence of the disease in communities increases (or decreases), further influencing infectious contact rates. This loop perpetuates the cycle of TB transmission, playing a central role in the dynamics of the TB-climate bio-socio-technical nexus.

**Table 2:** Effective feedback loops that reinforce the interactions of climate change and the incidence of TB*

Loop number	Title	Variables in loop
R1	Heatwave-energy use loop	Heatwave likelihood ↑ Air conditioner use rate↑ Electricity consumption rate↑ CO2 atmospheric level ↑ Heatwave likelihood
R2	Indoors time-airborne diseases risk loop	Average time spent indoors ↑ Indoor population density ↑ Ventilation level ↓ Airborne diseases risk ↑ Average time spent indoors↑
R3	Access-price loop	Food prices ↑ Food access ↓ Food prices ↑
R4	Malnutrition-infectious disease loop	Malnutrition risk ↑ Immune system strength ↓ Susceptibility to infectious diseases ↑ Nutrients absorption level ↓ Malnutrition risk ↑
R5	Health care cost-detection delay loop	Health care cost affordability ↓ Time to detection ↑ Infectious contact risk ↑Infection transmission risk ↑ Tuberculosis risk ↑ Work capacity (income) ↓ Health care cost affordability ↓
R6	Infectious contact-TB risk loop	Infectious contact risk ↑ (↓) Infection transmission risk ↑ (↓) Tuberculosis risk ↑ (↓) Infectious contact risk ↑

^*^(→ means leads to; ↑ means increase; ↓ means decrease)

These various feedback mechanisms are visually represented in the system map (Fig. 2), offering a comprehensive view of the complex dynamic interactions between TB and climate change. The interplay between these feedback loops and associated mechanisms shapes the evolving relationship between TB transmission, incidence, and the broader environmental context of climate change. Understanding these dynamics holistically is crucial for anticipating and preparing for the complex effects of imminent climate change on global TB patterns.

### Leverage points

Within this system map, certain leverage points emerge—key areas where targeted interventions could disrupt reinforcing feedback loops or enhance balancing ones. In the context of the TB-climate relationship, identifying and strategically acting on these points can help mitigate the risks posed by climate change.

## Discussion

To achieve a deeper and more practical understanding of the relationship between TB and climate change, we employed system mapping using a CLD approach. This visualization tool helps elucidate how various mechanisms, influenced by climate change, interact and contribute to TB transmission patterns. Based on findings from a systematic review and an expert validation session, the CLD highlights six reinforcing feedback loops that, if left unchecked, could significantly worsen the TB burden as climate change intensifies.

Our findings reveal that climate change—through heatwaves, cold spells, air pollution, and dust particles—leads to prolonged indoor confinement, especially for vulnerable populations. This confinement results in overcrowded indoor spaces, inadequate ventilation, and increased close contact, all of which elevate TB transmission risks. Ironically, health policies that recommend vulnerable groups, such as the elderly, pregnant women, and individuals with pre-existing conditions, to remain indoors during extreme temperature events may inadvertently contribute to increased TB transmission. Poor ventilation compounds this risk. For example, Nardell et al. demonstrated that when windows were closed and air conditioning was used, indoor CO₂ levels increased from 600 to 1600 PPM, indicating poor ventilation and a higher likelihood of airborne pathogens remaining suspended, thus amplifying TB exposure [48].

In addition, the increased reliance on air conditioning due to global warming presents another layer of complexity. While air conditioning addresses immediate comfort needs, it leads to higher electricity consumption, particularly in regions dependent on fossil fuels. This increases CO₂ emissions and forms a reinforcing feedback loop (R1), where rising temperatures drive greater energy use, further exacerbating global warming. The cycle of higher energy consumption, increased CO₂ emissions, and extended indoor confinement heightens TB transmission risks in poorly ventilated, densely populated spaces.

### Leverage points for intervention

To mitigate the risks posed by climate change on TB trends, several leverage points within the identified system can be targets for interventions:

#### Ventilation and air quality

Improving indoor ventilation is crucial. Sustainable solutions, such as solar-powered ventilation or passive cooling, should be prioritized to balance the need for cooling without exacerbating climate change. Building designs that promote natural ventilation can also reduce the concentration of airborne pathogens like Mycobacterium tuberculosis. Public health campaigns should emphasize the importance of indoor air quality, especially in densely populated areas.

#### Energy efficiency and renewable energy

Reducing reliance on fossil fuels for cooling systems is another key leverage point. Policymakers should advocate for the use of renewable energy sources, such as solar or wind power, to meet the growing demand for electricity in a more sustainable manner. This can help disrupt the feedback loop driving global warming and, consequently, TB transmission.

#### Public health and education

Health policymakers can promote natural ventilation practices, personal hygiene, and other preventive measures to reduce TB risks during periods of prolonged indoor confinement. Increased public awareness around the need for adequate ventilation and health-promoting behaviours can help reduce TB transmission.

#### Malnutrition and migration

Another significant leverage point is food security, as malnutrition weakens the immune system and increases susceptibility to TB. Climate change-induced food insecurity, particularly in regions experiencing droughts or extreme temperature events, can exacerbate malnutrition. Rising food prices limit access to essential nutrients, especially among vulnerable populations. Malnourished individuals face higher risks of TB infection and, as climate-induced migration drives people to overcrowded urban slums, the risk of TB transmission intensifies. Addressing food insecurity through sustainable agriculture, nutrition support programs, and healthcare access in these high-risk areas can help mitigate the impact of climate change on TB.

#### HIV-TB nexus

The interaction between TB and HIV adds further complexity to the climate-TB relationship. Prolonged indoor confinement reduces sunlight exposure, which impairs the body's natural production of vitamin D, essential for immune function. This is particularly concerning for individuals with HIV, who are already more vulnerable to TB. Antiretroviral therapy (ART), while crucial for managing HIV, exacerbates vitamin D deficiency [68]. A combined approach—offering nutritional support and integrating HIV and TB care—can reduce the risk of TB outbreaks among this high-risk population, especially as climate change disrupts traditional living environments.

#### System-wide action

The core feedback loop (R6) in the system map illustrates that failure to identify and treat TB patients increases infectious contact, leading to greater transmission and more active TB cases. This underscores the importance of public health measures such as active case finding, systematic screening of high-risk groups, and the efficient implementation of the Directly Observed Treatment, Short-Course (DOTS) program. Recommended by the WHO, DOTS involves health workers directly supervising TB patients as they take their medication, ensuring adherence and minimizing the risk of drug resistance [69]. Ensuring access to timely TB diagnosis and treatment—particularly in resource-constrained regions—is critical for controlling TB transmission in the context of climate change.

Beyond the need to strengthen DOTS and disrupt R6, climate change also poses serious challenges to the preparedness and resilience of health systems. Extreme weather events, including floods and storms, can damage healthcare infrastructure and reduce patient access to essential services. The collapse of health systems during emergencies limits TB-related education, prevention, treatment, and continuity of care [70]. Such disruptions may undermine TB control programmes and contribute to the emergence of multidrug-resistant TB (MDR-TB) [71, 72]. To meet these challenges, health systems must be strengthened to withstand climate shocks. This includes bolstering infrastructure, developing emergency response plans, training healthcare workers in crisis management, and safeguarding medicine and medical supply chains. Additionally, modern technologies—such as health information systems—can enhance coordination and responsiveness during crises. Implementing these strategies will help ensure continuous, high-quality TB care in an increasingly unpredictable climate.

Building on these systemic vulnerabilities, TB control policies must evolve into more comprehensive, climate-responsive strategies. While initiatives such as the WHO End TB Strategy, DOTS, and integrated HIV–TB care have significantly contributed to reducing TB incidence, they often overlook climate-related risks [73–75]. This is increasingly problematic, given that climate change is progressing at an accelerating and non-linear pace. Mechanisms that may appear marginal today—such as disrupted healthcare access or food insecurity—could rapidly escalate and undermine global TB control efforts [76]. Importantly, many high-burden countries—such as India, Indonesia, and the Philippines—are also highly vulnerable to climate impacts [3, 73]. Individuals living with TB are particularly at risk, as they are already affected by poverty, malnutrition, and comorbidities like HIV, all of which hinder their adaptive capacity. Without integrating climate resilience into TB policies, current strategies risk perpetuating health inequities and falling short of global elimination goals.

While many of the identified feedback loops present clear leverage points for intervention, certain climate-related variables—such as wind speed—remain contested in the literature. Some studies suggest that stronger winds enhance natural ventilation and help dilute airborne pathogens [46, 58], while others argue that wind can disperse Mycobacterium tuberculosis and air pollutants (e.g., PM10, SO₂) over larger areas, potentially increasing transmission risks [13, 31]. These divergent perspectives highlight the need for further empirical research to clarify the role of wind in shaping TB dynamics in a warming climate.

Given the complexity and interdependence of these factors, effective intervention will require systemic solutions that address multiple leverage points concurrently. Measures such as improving ventilation, strengthening food security, promoting renewable energy, and expanding public health education must be integrated into broader policy frameworks. Additionally, the intersecting vulnerabilities associated with HIV, migration, and poverty underscore the importance of a coordinated, multi-sectoral response.

At present, there is no coherent global strategy to guide countries in addressing the combined impacts of TB and climate change. This represents a significant gap in global health governance. Developing an evidence-based, consensus-driven framework that integrates climate resilience into TB control efforts would not only address an urgent emerging threat but also serve as a timely opportunity to reinforce and future-proof global health policy.

### Limitations

While the health impacts of climate change are increasingly recognized in the literature, few studies have directly examined its influence on TB. To address this gap, our study applied broad and flexible inclusion criteria to capture any articles that referenced potential mechanisms linking climate change to pulmonary TB. This breadth, while necessary for conceptual mapping, introduced substantial heterogeneity in exposures and outcomes, making quantitative synthesis (e.g., meta-analysis) infeasible.

Another limitation lies in the qualitative nature of our approach. While the study identifies plausible pathways through which climate change may affect TB transmission, it does not assess the relative causal contribution of each mechanism. As such, the results should be interpreted as conceptual guidance rather than precise empirical evidence.

Furthermore, due to the constraints of a systematic review, we could not control for confounding variables such as seasonality, socio-economic changes, or access to healthcare services. For example, previous research suggests that TB incidence peaks in spring and summer [77–80], potentially due to poor ventilation and crowding during winter months. Concurrently, wintertime spikes in environmental pollutants (e.g., SO₂, NO₂, O₃, CO) [81–83] may also contribute to delayed TB activation, a pattern echoed in our system map. Future studies should investigate these interactions more deeply and explore confounding influences.

Finally, while our study provides a foundational system map of climate–TB linkages, future research should pursue simulation-based approaches. Methods such as system dynamics modelling can help quantify causal pathways, assess intervention strategies, and support data-driven public health planning.

## Conclusion

This study has explored how climate change may affect tuberculosis (TB) patterns through a network of interconnected mechanisms. As the world strives to eliminate TB by 2050 [11, 84], it is increasingly important to recognise climate change as a critical threat—one that could undermine current progress if left unaddressed. The complexity and dynamic nature of this relationship suggest that a narrow focus on individual or well-known drivers is insufficient. Overlooking the subtler feedback loops and less obvious pathways identified in this study risks oversimplifying the problem and designing ineffective interventions.

Our findings highlight the need for health policies to adopt a more systemic and nuanced approach. Considering the multifaceted interactions—such as those involving ventilation, migration, malnutrition, and air pollution—can enable the development of more targeted, integrated, and forward-looking interventions.

Given the mounting evidence of climate change’s impact on public health, policymakers must act decisively. This includes supporting long-term studies to track trends, developing predictive models to enhance preparedness, and embedding climate-responsive actions—such as promoting renewable energy, improving indoor air quality, and addressing food insecurity—into TB control strategies.

Crucially, future efforts must account for the disproportionate burden borne by vulnerable and at-risk populations. A coordinated, cross-sectoral response—linking health, environment, housing, agriculture, and climate policy—is essential to address these overlapping vulnerabilities.

In sum, managing TB in the era of climate change will require a holistic and collaborative strategy. Such an approach is not only necessary for mitigating current risks but will be key to keeping global TB elimination efforts on track.

## Supplementary Information

Additional file 1: Search strategy used in three major databases (PubMed, Web of Science, and Scopus). This table provides an overview of the search strategy employed across the three primary academic databases. It outlines the specific search terms, Boolean operators, and filters applied to identify relevant studies related to climate change and TB transmission. The table presents the records retrieved for each search query at the time of conducting the research and highlights the combination of keywords used to refine the results. Additionally, a summary section reports the total number of records, the duplicates identified, and the final dataset used for analysis

## Data Availability

No datasets were generated or analysed during the current study.
