# Supplementary material for: Understanding the mechanisms of climate change impact on tuberculosis: a complex systems approach
Source: BMC Public Health. 2025 Oct 8;25:3382. doi: 10.1186/s12889-025-24709-6 (PMC12506164; doi:10.1186/s12889-025-24709-6)
Supplement: Supplementary file 1 — Additional file 1: Search strategy used in three major databases (PubMed, Web of Science, and Scopus). This table provides an overview of the search strategy employed across the three primary academic databases. It outlines the specific search terms, Boolean operators, and filters applied to identify relevant studies related to climate change and TB transmission. The table presents the records retrieved for each search query at the time of conducting the research and highlights the combination of keywords used to refine the results. Additionally, a summary section reports the total number of records, the duplicates identified, and the final dataset used for analysis [file 12889_2025_24709_MOESM1_ESM.docx]

Supplementary Table 1. Search strategy used in three major databases

| **PubMed** | | |
| --- | --- | --- |
| **Time: 12:24:19**  **Sep /14/2024** | | |
| #1 | Climate Change [MeSH Terms] | 34.406 |
| #2 | Climate Change [Text Word] | 78.102 |
| #3 | global climate changes [Text Word] | 330 |
| #4 | global warming [Text Word] | 17.614 |
| #**5** | weather changes [Text Word] | 367 |
| #6 | #1 OR #2 OR #3 OR #4 OR #5 | 89.941 |
| #7 | communicable diseases [MeSH Terms] | 385.038 |
| #8 | communicable diseases [Text Word] | 57.349 |
| #9 | infectious disease [Text Word] | 91.589 |
| #10 | contagious disease [Text Word] | 2.084 |
| #11 | transmissible diseases [Text Word] | 747 |
| #12 | Mycobacterium tuberculosis [MeSH Terms] | 59.531 |
| #13 | Mycobacterium tuberculosis [Text Word] | 78.698 |
| #14 | Tuberculosis [MeSH Terms] | 210.560 |
| #15 | Tuberculosis [Text Word] | 287.084 |
| #16 | M. tuberculosis [Text Word] | 21.697 |
| #17 | TB [Text Word] | 77.876 |
| #18 | #7 OR #8 OR #9 OR #10 OR #11 OR #12 OR #13 OR #14 OR #15 OR #16 OR #17 | 951.343 |
| #12 | #6 AND #18 | 1.517 |
| **Web of Science** | | |
| **Time: 12:39:34**  **Sep 14 2024** | | |
| #1 | TS= (Climate Change) | 555,027 |
| #2 | TS= (weather changes) | 75,843 |
| #3 | TS= (global climate changes) | 155,219 |
| #4 | TS= (global warming) | 101,810 |
| #5 | #1 OR #2 OR #3 OR #4 | 642,263 |
| #6 | TS= (communicable diseases) | 22,554 |
| #7 | TS= (infectious disease) | 216,239 |
| #8 | TS= (contagious disease) | 10,828 |
| #9 | TS= (transmissible diseases) | 8,913 |
| #10 | TS= (Mycobacterium tuberculosis) | 85,876 |
| #11 | TS=(tuberculosis) | 182,687 |
| #12 | TS= (M. tuberculosis) | 32,576 |
| #13 | TS=(TB) | 108,484 |
| #14 | #6 OR #7 OR #8 OR #9 OR #10 OR #11 OR #12 OR #13 | 475,055 |
| #15 | #5 AND #14 | 4.188 |
| **Scopus** | | |
| **Time: 12:52:34**  **Sep 14 2024** | | |
| #1 | TITLE-ABS-KEY (climate change) | 624,780 |
| #2 | TITLE-ABS-KEY (weather changes) | 77,243 |
| #3 | TITLE-ABS-KEY (global climate changes) | 185,491 |
| #4 | TITLE-ABS-KEY (global warming) | 139,487 |
| #5 | (TITLE-ABS-KEY climate change) OR (TITLE-ABS-KEY (weather changes)) OR (TITLE-ABS-KEY (global climate changes)) OR (TITLE-ABS-KEY (global warming)) | 737,600 |
| #6 | TITLE-ABS-KEY (communicable diseases) | 101,635 |
| #7 | TITLE-ABS-KEY (infectious disease) | 387,037 |
| #8 | TITLE-ABS-KEY (contagious disease) | 17,220 |
| #9 | TITLE-ABS-KEY (transmissible diseases) | 13,956 |
| #10 | TITLE-ABS-KEY (Mycobacterium tuberculosis) | 123,971 |
| #11 | TITLE-ABS-KEY (tuberculosis) | 375,395 |
| #12 | TITLE-ABS-KEY (M. tuberculosis) | 37,835 |
| #13 | TITLE-ABS-KEY (TB) | 128,407 |
| #14 | (TITLE-ABS-KEY (communicable diseases)) OR (TITLE-ABS-KEY (infectious disease)) OR (TITLE-ABS-KEY (transmissible diseases)) OR (TITLE-ABS-KEY (contagious disease)) OR TITLE-ABS-KEY (Mycobacterium tuberculosis OR (TITLE-ABS-KEY (tuberculosis) OR TITLE-ABS-KEY (M. tuberculosis) OR(TITLE-ABS-KEY(TB) | 128,407 |
| #15 | (TITLE-ABS-KEY climate change) OR (TITLE-ABS-KEY (weather changes) OR (TITLE-ABS-KEY (global climate changes) OR (TITLE-ABS-KEY (global warming)) AND ((TITLE-ABS-KEY (communicable diseases) OR ( TITLE-ABS-KEY ( infectious disease ) OR ( TITLE-ABS-KEY ( transmissible diseases ) OR ( TITLE-ABS-KEY ( contagious disease ) OR (TITLE-ABS-KEY(Mycobacterium tuberculosis) OR (TITLE-ABS-KEY(tuberculosis) OR TITLE-ABS-KEY(M. tuberculosis) OR(TITLE-ABS-KEY(TB)) | 5,881 |

| **Total (3 databases)** | **11586** |
| --- | --- |
| **duplicates** | **5257** |
| **final** | **6329** |

**Description**: The search strategy includes three main databases (PubMed, Web of Science, and Scopus). The table provides an overview of the search strategy employed across the three primary academic databases. It outlines the specific search terms, Boolean operators, and filters applied to identify relevant studies related to climate change and tuberculosis transmission. The table presents the records retrieved for each search query at the time of conducting the research and highlights the combination of keywords used to refine the results. Additionally, a summary section reports the total number of records, the duplicates identified, and the final dataset used for analysis.
